# Differences in Colorectal Cancer Survival Based on Primary Tumor Location: Retrospective Study from a Single Institution

**DOI:** 10.7150/jca.85695

**Published:** 2023-08-06

**Authors:** Manuel Diez-Alonso, Fernando Mendoza-Moreno, Miguel A. Ortega, Hector Aguado, Belen Matías, Cristina Vera, Sonia Soto, Ana Quiroga, Silvestra Barrena Blazquez, Melchor Alvarez de Mon, Alberto Gutierrez-Calvo

**Affiliations:** 1 Department of General and Digestive Surgery, University Hospital Príncipe de Asturias, 28805 Madrid, Spain.; 2 Departamento de Cirugia, Ciencias Medicas y Sociales. Faculty of Medicine and Health Sciences, University of Alcalá, 28801 Alcalá de Henares, Spain.; 3 Department of Medicine and Medical Specialities, Faculty of Medicine and Health Sciences, University of Alcalá, 28801 Alcalá de Henares, Spain.; 4 Ramón y Cajal Institute of Sanitary Research (IRYCIS), University Hospital Príncipe de Asturias, 28034 Madrid, Spain.; 5 Department of General and Digestive Surgery. Surgical Nurse. University Hospital Príncipe de Asturias, 28805 Madrid, Spain.; 6 Immune System Diseases-Rheumatology and Internal Medicine Service, University Hospital Príncipe de Asturias, (CIBEREHD), 28806 Alcalá de Henares, Spain.

**Keywords:** colorectal cancer, survival, tumor recurrence, overall survival, time to recurrence, cancer-related survival, relapse-free survival

## Abstract

**Objective:** The location of the primary tumor in colorectal cancer (CRC) could be a prognostic factor related to survival. However, its usefulness has not been sufficiently analyzed. The results in patients with tumors in initial stages are very limited, and there are descriptive parameters of survival that have not been analyzed in detail. In this study, the relationship between primary tumor location and survival in CRC patients was analyzed.

**Materials And Methods:** This was a retrospective observational study. All patients treated consecutively for CRC between January 2005 and December 2019 in the same hospital center were included. Overall survival (OS), cancer-related survival (CRS), time to recurrence (TTR), relapse-free survival (RFS) and postrecurrence survival (PRS) were analyzed, and the results were classified by tumor stage. The results were compared among patients with right colon (RS), left colon (LS) and rectal tumors.

**Results:** In the entire cohort, patients with RS tumors had lower OS and lower CRS at 60 months after diagnosis than did patients with LS or rectal tumors. In the regression analysis, the localization of the primary tumor was an independent prognostic indicator for OS and CRS. Analysis by tumor stage showed that patients with RS stage III tumors had lower OS and lower CRS at 60 months than did patients with LS and rectal tumors (42%, 59% and 53%, respectively, p = 0.006; and 48%, 63% and 57%, respectively, p = 0.025). Additionally, patients with RS Stage IV tumors had lower OS and lower CRS at 36 months than did patients with LS and rectal tumors (9%, 24%, 24%, respectively, p < 0.001; and 10%, 24% and 24%, respectively, p < 0.001). No differences were found in TTR and RFS among patients with stage I and II RS, LS, and rectal tumors. In contrast, patients with stage RS III tumors had significantly poorer PRS (9% for RS tumors, 13% for LS tumors, and 22% for rectal tumors) (p < 0.001).

**Conclusion:** The location of the primary tumor in patients with CRC is related to survival. The effect of laterality is more marked in patients with stage III and IV tumors. Patients with RS tumors had lower OS and CRS due to the lower survival of patients with stage IV RS tumors and lower PRS for patients with stage III tumors.

## Introduction

In recent years, primary tumor location has been included among the prognostic factors in patients with adenocarcinoma of the colon and rectum (CRC). Various publications have shown that this variable may be related to the survival of patients with this type of tumor 1-5. For patients with stage IV tumors originating in the right colon, survival is lower than that for patients with tumors originating in the left colon and rectum; this effect is independent of the chemotherapy treatment followed 5-8. This may be the result of interactions among multiple factors. There are clinical, histopathological and molecular differences between tumors of the right colon and tumors of the left colon; however, the weight of the relative influence of each of these differences on the effect of laterality on CRC survival is not known.

The relationship between primary tumor location and the prognosis of CRC has not been sufficiently analyzed. The effect of laterality in patients with early-stage CRC has not been studied in detail. It is possible that primary tumor location does not have the same weight in each of the tumor stages. A meta-analysis found greater overall survival for patients with tumors in initial tumor-node-metastasis (TNM) stages in the left colon than in patients with tumors in initial TNM stages originating in the right colon (hazard ratio, 0.82; 95% confidence interval, 0.79-0.84; P < 0.001) 1. However, in this publication, the analysis was performed by grouping all the tumor stages. Studies based on data from population registries, such as the “Surveillance, Epidemiology, and End Results” (SEER) program, and several studies with data from other registries 9-17 also evaluated the effect of primary tumor location on CRC. The results for stages I, II and III tumors provided disparate results, with either no difference or improved OS outcomes with RS tumors. Furthermore, relapse-free survival was not evaluated in these studies.

Kerr analyzed the effect of laterality on OS, RFS and postrecurrence survival in patients with stage II and III tumors using data obtained in the VICTOR and QUASAR2 clinical trials 18. These clinical trials were designed to assess the efficacy of rofecoxib 19 and capecitabine plus bevacizumab 20 in the adjuvant setting of CRC. No relationship was found between primary tumor location and RFS, although patients with tumors of the right colon showed lower OS 18. The authors attributed this difference to the effect of primary tumor location on postrecurrence survival.

In many studies, the end-points analyzed for survival have been insufficient. Studying only OS and RFS can provide partial information because both parameters include all types of deaths, related or not to CRC 21. Furthermore, TTR and PRS have been analyzed in very few studies 16,18. In this study, we analyzed the effect of tumor laterality on the survival of patients with CRC. For this, overall survival (OS), cancer-related survival (CRS), time to recurrence (TTR), relapse-free survival (RFS) and postrecurrence survival (PRS) were analyzed, and the results were classified by tumor stage.

## Patients and Methods

This was a retrospective observational study. All patients treated for colorectal adenocarcinoma between January 2005 and December 2019 in the General Surgery Department of Hospital Universitario Príncipe de Asturias, Alcala de Henares, Madrid, Spain, were included. The main objective of the study was to analyze the relationship between primary tumor location and the survival of patients with CRC. Survival was determined for all patients who met the inclusion criteria, and the results were compared among patients with tumors of the right colon, left colon, and rectum. The study adhered to the STROBE guidelines for designing and reporting observational studies. Patients were identified from the computerized database of the Coloproctology Unit that has been prospectively developed over the years. The study was approved by the Ethics Committee of Hospital Príncipe de Asturias (Code: OE 37/2021).

### Patients and data

**Figure 1:**
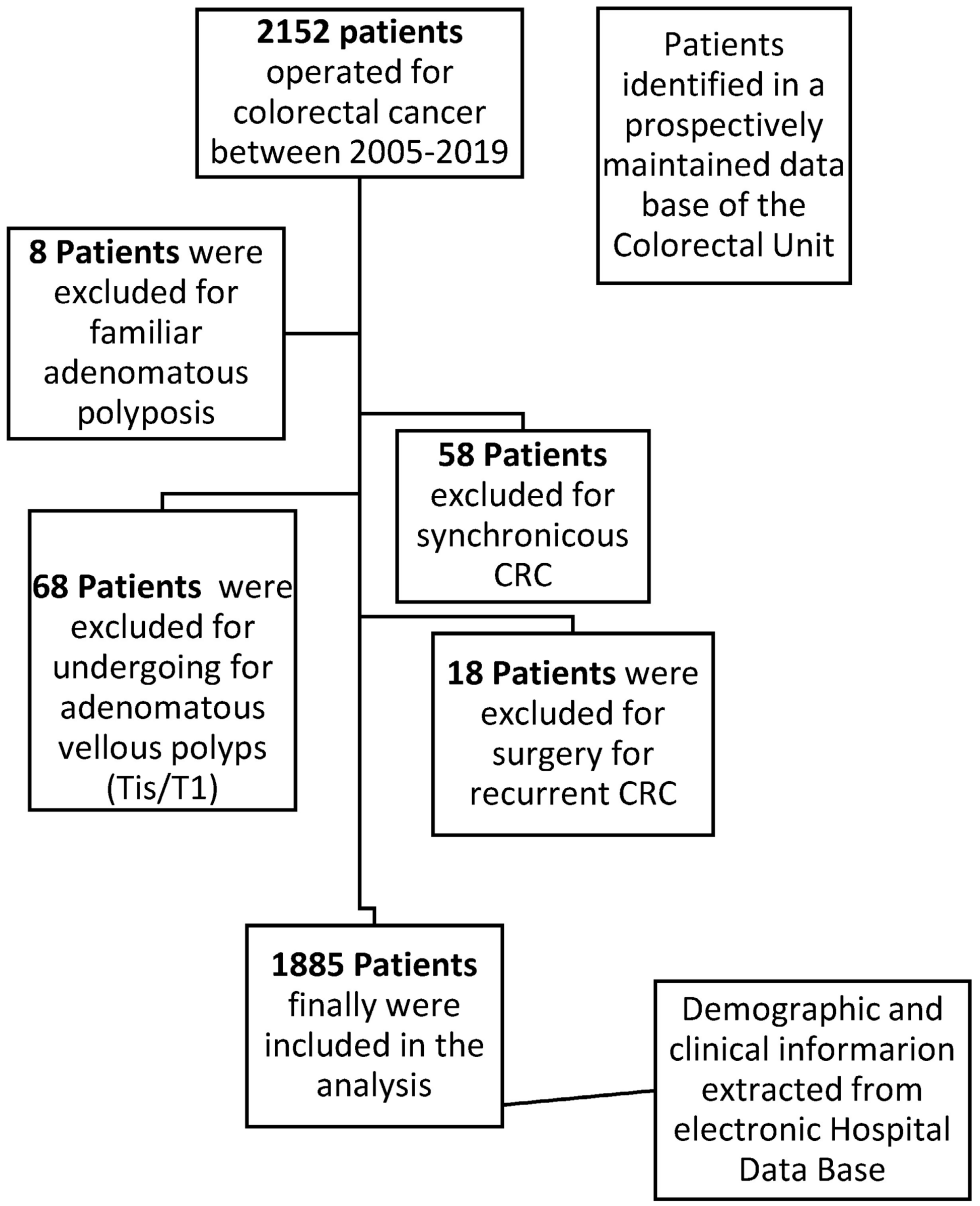
Flowchart detailing the selection of the patients in this study.

The inclusion criteria were as follows: age over 18 years and histopathology of the primary tumor compatible with colorectal adenocarcinoma. The exclusion criteria were familial multiple polyposis, synchronous tumors of the colon or rectum, history of previous cancer, adenomatous polyp or tumor in situ, and mucinous appendicular tumors (Figure 1). After the diagnosis of CRC, all patients were evaluated by a multidisciplinary medical committee that assessed the possible therapeutic options depending on the grade of extension, coexistence of metastasis in other organs, presence of local complications produced by the tumor and the functional status of the patient.

The clinical data of the patients were obtained from the electronic medical records of the hospital. Data related to coincident predictor variables were collected: demographics (sex and age), location of the primary tumor, histopathology (degree of differentiation, histological type, mucinous component, presence and number of adenopathies, and degree of local tumor infiltration), presence of metastases distance, surgical procedures, postoperative complications, medical oncological treatment received and long-term outcome. CRC was staged using the TNM classification of the American Joint Committee on Cancer (AJCC). There were no missing data for any of the variables that were included in the analysis.

For this study, tumors located in the cecum, ascending colon, hepatic angle and transverse colon were classified as right-sided tumors (RS); those originating in the splenic flexure, descending colon and sigma were classified as left-sided tumors (LS); and those located in the proximal 15 cm of the anus were classified as rectal tumors. Patients were stratified into three groups based on age: <50 years, 50-69 years, and 70 years or older.

After the initial treatment, the patients were followed up in consultations in accordance with the current guidelines by means of physical examinations, analytical assessments every six months during the first two years and then annually; annual computed tomography scans up to the fifth year; and colonoscopy 1 and 3 years after surgery.

### Main outcome measures

The primary outcomes of interest were OS and cancer-specific survival (CSS). For OS, deaths due to any cause were considered. Survival was estimated in months from the date of diagnosis to the last date of follow-up or date of death for nonsurvivors. For CSS, deaths due to CRC were considered deaths, and those due to another cause were censored. The secondary outcomes of interest were relapse-free survival (RFS) (time between diagnosis of CRC and tumor relapse or death due to any cause), TTR (time from diagnosis to the time of recurrence; patients with no disease recurrence were censored at the last time at which they were known to be recurrence free), and post recurrence survival (PRS) (time from the date of diagnosis of recurrence to the time of death).

### Statistical analysis

The variables were input into a Microsoft Excel 2019 (v.27) (Microsoft, Redmond, WA, USA) spreadsheet. Statistical analysis was performed using SPSS (v.23) (IBM, Armonk, New York, NY, USA).

Initially, the distribution of patient and tumor characteristics among the RS, LS, and rectal tumor groups was compared using the x-squared test. Next, survival up to 60 months after diagnosis and median survival for each variable included in the present study were analyzed using the Kaplan‒Meier estimator. For patients with stage IV tumors, survival was calculated at 36 months after the intervention because the number of patients who survived at 60 months was very small. OS and CRS were studied in the entire cohort of patients. For the analysis of RFS and PRS, only patients with stage I, II and III tumors were included. The log-rank test was used to compare survival curves.

Finally, the effect of each variable on survival was evaluated using Cox proportional hazard regression. Cox regression models were built using the backward method. Variables included in the adjusted models had a p value <0.05 for the outcome of interest in the univariate analysis. These variables remained in the final model if they were still significant at p<0.05 in the final adjusted model. The assumption of proportional hazards across different covariates was tested by inspect the log (-log) plots. The risk of death or recurrence was expressed as the hazard ratio (HR) and 95% confidence interval (CI).

## Results

A total of 1885 patients met the criteria to be included in the analysis. The tumor was located in the right colon in 609 (32.2%) patients, in the left colon in 766 (40.6%) patients and in the rectum in 510 (20.1%) patients. The mean age of the sample was 68 ± 12 years. Among them, 379 (20.1%) had stage I tumors, 615 (32.6%) had stage II tumors, 469 (24.9%) had stage III tumors, and 422 (22.4%) had stage IV tumors.

Table 1 shows the distribution of the clinical and histopathological characteristics in the three groups of patients. In the group with right colon tumors, there was a greater proportion of women (43.5%; p = 0.008), patients older than 70 years (56.81%; p < 0.001), T4 tumors (21.2%; p < 001), N2 tumors (20.2%; p = 0.029), mucinous tumors (20%; p < 0.001), and tumors with poor grade of differentiation (17,24%; p<0.001). The distribution of the categories of the TNM Stage were similar in the three group of patients.

### Survival

#### Entire cohort

Kaplan-Meier estimations of OS and CRS at 60 months after diagnosis for the entire cohort were 53% and 60%, respectively.

Patients whose primary tumor was located in the right colon had lower OS at 60 months after diagnosis than did those whose primary tumor was located in the LS and rectum (49%, 56% and 55% in RS, LS and rectum, respectively) (p = 0.011) (Figure 1). The results of the univariate survival analysis are provided in Supplemental Table 1, including all the clinical and histopathological variables analyzed. In the Cox multiple regression analysis (Table 2), primary tumor locations was an independent predictor of OS. The risk associated with RS tumors was higher than that associated with LS tumors (HR: 0.679; 95% CI: 0.511-0.902; p = 0.042), and the risk associated with LS tumors was higher than that associated with rectal tumors (HR: 0.737; 95% CI: 0.572-0.949; p = 0.018). The other factors that were independent predictors were tumor intestinal obstruction (p < 0.001), tumor perforation (p < 0.001), T stage (p < 0.001), N stage (p < 0.001), tumor grade of differentiation (p = 0.032), age (p < 0.001), and postoperative intraabdominal infection (p = 0.033).

Similarly, patients with RS tumors had lower CRS at 60 months after diagnosis than did patients with LS tumors and rectal tumors (56%, 62% and 62%, respectively) (p = 0.020). For CRS, Cox multiple regression analysis showed that the independent prognostic value of laterality was not statistically significant (p = 0.234). The factors with independent predictive value were obstruction (p < 0.001), perforation (p < 0.001), T stage (p < 0.001), N stage (p < 0.001), tumor grade of differentiation (p = 0.035), and age (p < 0.001) (Table 2).

#### Survival by TNM Stage

*Stage I.* For stage I tumors, patients with RS, LS and rectal tumors had the same OS (84%) at 60 months after diagnosis (p = 0.959). Additionally, CRS was similar among the three locations: 96% for RS, 94% for LS and 90% for rectum tumors (0.333). The multivariate analysis results for stage I tumors are shown in Supplemental Table 2S.

*Stage II*. Patients with RS tumors had lower OS at 60 months after diagnosis than did patients with LS tumors and rectal tumors; however, the difference was not statistically significant (66%, 73% and 70%, respectively) (p = 0.378). Similarly, CRS was lower for patients with RS tumors but not statistically significant (79%, 82% and 85%, respectively) (p = 0.283). The multivariate analysis results for stage II tumors are shown in Supplemental Table 3S.

*Stage III*. For stage III tumors, OS was significantly lower for patients with RS tumors than for patients with LS tumors and rectal tumors (42%, 59% and 53%, respectively) (p = 0.006) (Figure 3). Multiple regression analysis indicated that laterality was an independent predictor of OS (Table 3). The risk associated with RS tumors was higher than that associated with LS tumors (HR: 0.991; 95% CI: 0.686-1-433; p = 0.963), and the risk associated with LS tumors was higher than that associated with rectal tumors (HR: 0.653; 95% CI: 0.461-0.926; p = 0.017).

Additionally, CRS for patients with RS tumors (48%; median 44 months) was lower than that for patients with LS and rectal tumors (63% and 57%, respectively) (median not reached) (p = 0.025). Multiple regression analysis revealed that laterality had independent prognostic value. The risk associated with RS tumors was higher than that associated with LS tumors (HR: 0.932; 95% CI: 0.630-1.380; p = 0.725), and the risk associated with LS tumors was higher than that associated with rectal tumors (HR: 0.642; 95% CI: 0.443-0.932: p = 0.020).

**Figure 2:**
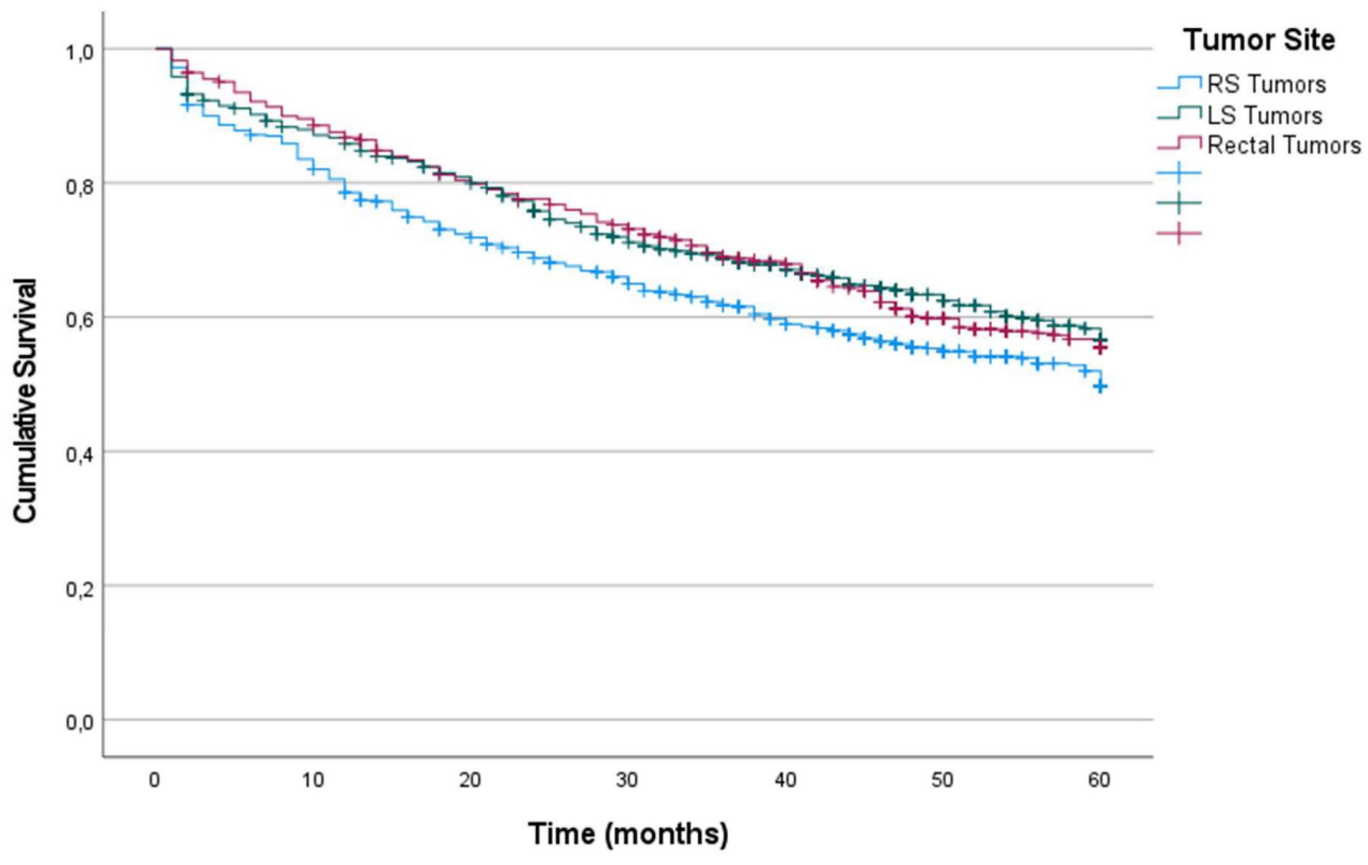
Kaplan-Meier estimates of Overall Survival for the entire cohort according to localization of primary tumor.

**Figure 3:**
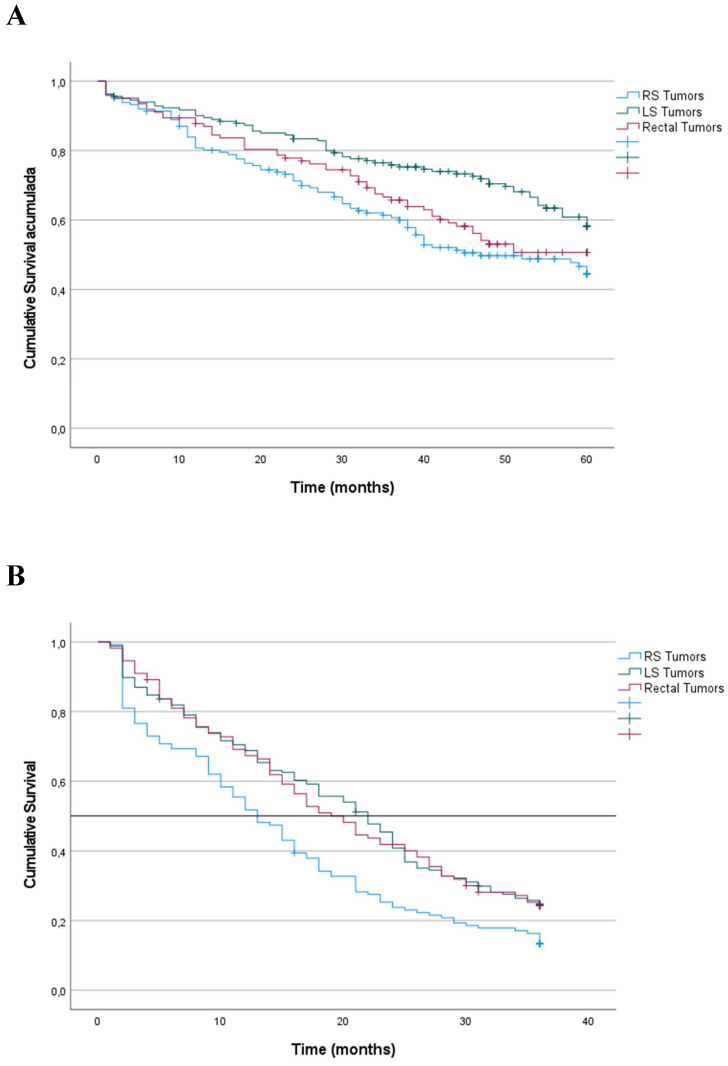
Kaplan‑Meier survival estimates of OS according to localization of primary tumor. **A.** Patients with TNM Stage III, OS at 60 months after diagnosis. **B.** Patients with TNM Stage IV, OS at 36 months after diagnosis. Horizontal bar denotes median survival.

*Stage IV*. For patients with stage IV tumors, survival was calculated at 36 months after the intervention. OS for patients with RS tumors was significantly lower than that for patients with LS (9%; median 13 months) than LS tumors (24%; median 22 months), rectal tumors (24%; median 20 months) (p < 0.001) (Figure 2). Multiple regression analysis revealed that laterality was an independent predictor of OS (Table 3). The risk associated with RS tumors was higher than that associated with LS (HR: 0.679; 95% CI: 0.511-0.902; p = 0.007), and the risk associated with LS tumors was higher than that associated with rectal tumors (HR: 0.737; 95% CI: 0.572-0.949; p = 0.007).

Additionally, CRS was significantly lower for patients with RS tumors than LS tumors and rectal tumors (10%; median 13 months), than LS tumors (24%; median 22 months) and rectal tumors (24%; median 20 months) (p < 0.001). Primary tumor location was an independent predictor of CRS. The risk associated with RS tumors was higher than that associated with LS tumors (HR, 0.691; 95% CI, 0.520-0.918), and the risk associated with LS tumors was higher than that associated with rectal tumors (HR, 0.751; 95% CI, 0.583-0.968).

### Recurrence

#### Entire Cohort

Among the 1,461 patients with stages I, II and III tumors at the time of diagnosis, tumor recurrence was detected in 326 (22.3%). Kaplan-Meier estimates for the entire cohort for TTR, RFS, and PRS at 60 months of follow-up were 73%, 65%, and 16%, respectively. There were 24 (6.3%) patients with stage I tumors, 106 (17.2%) with stage II tumors and 196 (41.8%) with stage III tumors. Based on primary tumor location, 99 (20.9%) patients had RS tumors recurrence, 122 (20.7%) had LS tumor recurrence, and 105 (26.3%) had rectal tumor recurrence (p = 0.079). Supplemental Table 5S shows the univariate analysis results for TTR and RFS, including all the clinical and histopathological variables analyzed.

There were no statistically significant differences in TTR and RFS among the three tumor sites. The TTR was 74% for patients with RS tumors, 76% for patients with LS tumors, and 70% for patients with rectal tumors (p = 0.188). RFS was 63% for patients with RS tumors, 68% for patients with LS tumors and 64% for patients with rectal tumors (p = 0.302).

**Figure 4:**
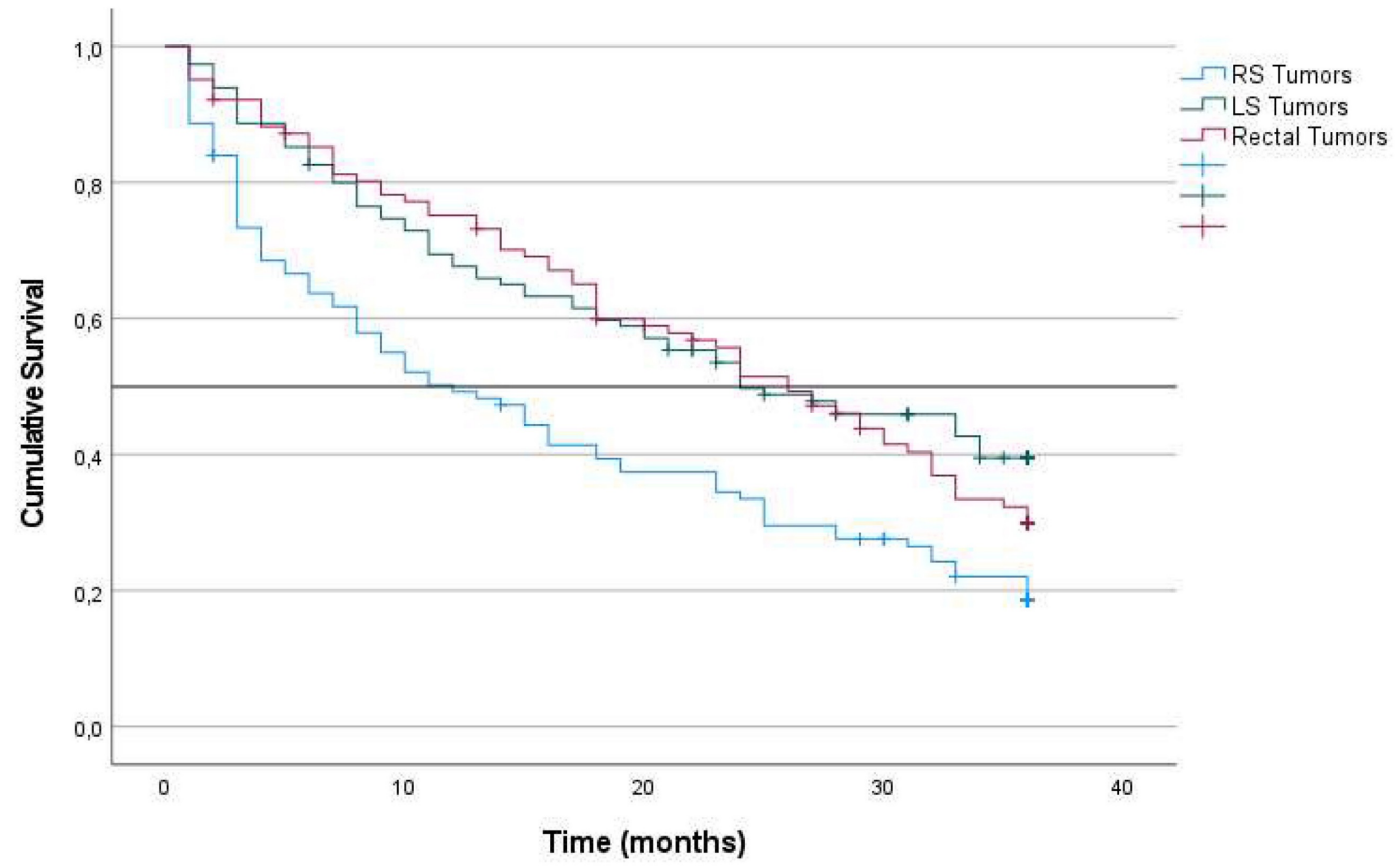
Kaplan‑Meier survival estimates of PRS at 36 months after recurrence according to localization of primary tumor. Horizontal bar denotes median survival.

In contrast, PRS was significantly lower for patients with RS tumors (9%; median 12 months), LS tumors (13%; median 25 months) and rectal tumors (22%; median 26 months) (p < 0.001) (Figure 4). Additionally, Cox multiple regression analysis revealed that laterality was an independent predictor of PRS (Table 4). The risk associated with RS tumors was higher than that associated with LS tumors (HR, 0.709; 95% CI, 0.512-0.980; p = 0.037), and the risk associated with LS tumors was higher than that associated with rectal tumors (HR, 0.644; 95% CI, 0.475-0.875; p = 0.005).

In addition, among patients with tumor recurrence, OS was lower for patients with RS tumors (13%; median 32 months) than for patients with LS tumors (32%; median 50 months) and for patients with rectal tumors (36%; median 47 months) (p ≤ 0.001). Similarly, CRS was lower for patients with RS tumors than for patients with LS tumors and rectal tumors (15%, 32% and 36%, respectively) (p < 0.001).

#### Relapse-free survival by TNM stage

In patients with stage I and stage II tumors, there were no significant differences among the three locations with regard to TTR, RFS and PRS (Table 5).

For stage III tumors, patients with RS tumors had lower PRS (6%; median 10 months) than did patients with LS tumors (14%; median 28 months) and patients with rectal tumors (15%; median 24 months) (p = 0.002) (Table 5). TTR and RFS were not significantly different among the three tumor locations (Table 5).

## Discussion

The results of this study indicate that primary tumor location is related to the survival of patients with CRC. In the entire cohort, patients whose primary tumor was located in the right colon had lower OS and lower CRS at 60 months after diagnosis than did those whose primary tumor was located in the LS and rectum. In addition, Cox multiple regression analysis, revealed that primary tumor location was an independent predictor of OS and CRS. To further investigate the relationship between tumor location and survival, the results were analyzed by tumor stage. OS and CRS were lower for patients with stage III and stage IV RS tumors only.

Both the frequency of recurrence, RFS and TTR were similar among stages I, II and III tumors in the three locations. However, survival after a diagnosis of recurrence was significantly lower for patients with RS tumors, in particular those with stage III tumors (PRS at 60 months: 6% for patients with RS tumors, 11% for patients with LS tumors and 16% for patients with rectal tumors; p = 0.006). Cox multiple regression analysis revealed that laterality was an independent predictor of PRS in patients with stage III disease. Furthermore, among patients with tumor recurrence, OS and CRS were lower for patients with RS tumors. These finding verify that the lower OS and CRS in the group of patients with RS tumors was due to the combination of the lower survival of patients with stage IV RS tumors and lower PRS for patients with stage III tumors.

It is difficult to compare the results of this study with those of other publications. A detailed analysis of the publications shows discrepancies in the results, stemming from both the origin of the data analyzed (clinical series vs. population registries) and the study design. In addition, some studies are based solely on patients with advanced tumors, and others include all types of patients without differentiating TNM stages. Long-term survival endpoints also vary from one study to another, and most only analyze OS, even though this parameter may be influenced by various coinciding factors, such as patient age and associated comorbidities 21.

Most publications conclude that among stage IV tumors, compared with LS and rectal tumors, RS tumors are associated with lower survival rates 5-8. The results of this study also support this hypothesis. In fact, primary tumor location has been proposed to be included in prognosis prediction models for patients undergoing surgical treatment for colorectal liver metastasis 9,21-23.

With respect to patients with tumors in early stages, two meta-analyses, one by Petrelli 1 and the other by Yahagi 15, evaluated outcomes based on primary location in 66 and 15 studies, respectively. Both found consistently improved OS outcomes with LS tumors than with RS tumors. However, in both publications, the analysis was performed by grouping tumor stages I, II and III. One of those studies concluded that tumors on the left side of the colon are significantly associated with an absolute 19% reduced risk of death 1. Two large population registry-based studies evaluated OS and reached the same conclusion 14,16. In contrast, two SEER series concluded that for patients with RS tumors, OS was improved for those with stage II disease but poorer for those with stage III disease 10,11. Additionally, in a Canadian population-based study, the authors found no significant difference in survival when comparing patients with RS and LS tumors and concluded that disease laterality was not associated with long-term OS or CRS when all stages were combined or when individual disease stages were examined separately 13. Warschkow conducted the largest reported SEER series of 91,416 patients 12. Through univariate analysis, that study found poorer OS and CRS for patients with RS tumors. After propensity score matching, the prognosis of patients with RS tumors was better overall. The better prognosis resulted from an improvement in OS for those with stage I and II tumors because no difference in OS was observed for patients with stage III tumors based on primary tumor side. In a study by Lee, patients with stage I and II RS tumors had higher TTR, and for patients with stage III tumors, TTR was lower for those with RS tumors; however, there was no influence on OS 16.

These aforementioned data lead to confusion on the subject. The variation in the interpretation of these data highlights the complexity of the effect of tumor side on early-stage disease and the limitations of only examining OS outcomes. In this study, there were no differences in OS and CRS for patients with stage I and II tumors. In these groups, the tumor burden was low, and only a small proportion of patients had recurrence and ultimately died of cancer. The relative contribution of noncancer-related deaths as RFS events and OS events is more substantial.

In this study, the main difference observed between tumor locations in patients with early-stage tumors was lower PRS for patients with RS tumors. Although the frequency of recurrence, TTR and RFS were similar for patients with RS, LS and rectal tumors, patients with RS tumors with recurrence had a shorter survival time after the diagnosis of recurrence than did patients with LS and rectal tumors. This parameter has only been investigated in two previous studies. In a study by Kerr 18, no relationship was found between primary tumor location and RFS; however, patients with tumors in the right colon had lower OS. The investigators attributed the finding to the influence of lower PRS for patients with RS tumors. In the study by Lee 16, when each stage was evaluated individually, the difference in PRS was observed only for patients with stage III tumors; the author concluded that the poorer OS and CRS survival outcomes were driven by poorer PRS for patients with RS. Our data coincide with those reported by these authors; we think that the lower PRS obtained for patients with RS tumors indicates that these tumors have a higher degree of biological aggressiveness.

The data obtained suggest that "primary tumor location" is a secondary predictive factor, an epiphenomenon. As verified, in the group of patients with right colon tumors, there was a higher proportion of factors associated with worse survival: patients older than 70 years, T4 tumors, N2 tumors and mucinous tumors. We think that laterality is possibly a marker that reflects the clinical significance of differences in genetic alterations that occur in the different segments of the colon and rectum. Genetic alterations such as KRAS gene mutations, hypermethylation of cytosine-phosphate-guanine (CpG) islands (CIMP-high), BRAF mutations and microsatellite instability due to mutations or hypermethylation of mismatch repair genes (MSI-high) occur more frequently In tumors originating in the right colon than in the other segments of the colon and rectum 7.

The limitations of this study are those derived from its retrospective nature. As the information gathered was in part obtained from hospital records, we could not obtain and include complete data from comorbidity and adjuvant chemotherapy, which are important variables influencing survival. Besides, it is important to consider the potential for detection bias or misclassification inherent to any retrospective study. In addition, we could not include any genetic factor, which are well recognized to be related with the biologic behaviour of CRC. In our hospital KRAS mutations are assayed only in Stage IV tumors and in patients with tumor recurrences, only if those patients are candidates to be treated with biologic agents. The study of microsatellite instability has been included in the routine protocols in the recent studies.

## Conclusion

The results of this study indicate that the primary tumor location in patients with CRC is related to survival and that the effect of laterality is more marked in patients with stage III and IV tumors. The prognostic weight of primary tumor location on OS and CRS was lower than that of T stage, N stage and age; however, laterality maintained its independent value in the regression analysis when adjusting for the effect of these variables, providing complementary information. Patients with RS tumors had lower OS and CRS, which was due to the lower survival of patients with stage IV tumors and to lower PRS for patients with stage III tumors.

## Supplementary Material

Supplementary tables.

## Figures and Tables

**Table 1:** Patient and tumor characteristics categorized by localization of the primary tumor.

	Total patients (n=1885)	Right Colon (n=609)	Left Colon (n=766)	Rectum (n=510)	P value
SEX					
Women	731 (38.8%)	265 (43.5%)	287 (37.5%)	178 (34.9%)	0.008
Men	1154 (61.2%)	344 (56.5%)	479 (62.5%)	332 (65.1%)	
AGE (years)					
<50	136 (7.22%)	43 (7.06%)	47 (6.13%)	46 (9.01%)	<0.001
50-69	819 (43.44%)	220 (36.13%)	356 (46.48%)	243 (47.65%)	
≥ 70	930 (49.34%)	346 (56.81%)	363 (47.39%)	221 (43.34%)	
INTESTINAL OBSTRUCTION					
Present	211 (11.2%)	82 (13.5%)	112 (14.6%)	17 (3.3%)	<0.001
TUMOR PERFORATION					
Present	107 (5.67%)	27 (4.43%)	69 (9.00%)	11 (2.15%)	<.001
T STAGE					
T1	180 (9.54%)	59 (9.7%)	62 (8.1%)	59 (11.5%)	<0.001
T2	262 (13.90%)	87 (14.3%)	82 (10.7%)	93 (18.2%)	
T3	1087 (57.67%)	334 (54.8%)	464 (60.7%)	288 (56.5%)	
T4	356 (18.89%)	129 (21.2%)	157 (20.5%)	70 (13.8%)	
N STAGE					
N0	1068 (56.65%)	329 (54.02%)	450 (58.75%)	289 (56.66%)	0.029
N1	498 (26.42%)	157 (25.78%)	211 (27.55%)	130 (25.50%)	
N2	319 (16.93%)	123 (20.20%)	105 (13.70%)	91 (17.84%)	
TNM STAGE					
I	379 (20.1%)	126 (20.7%)	121 (15.8%)	132 (25.8%)	<0.001
II	615 (32.6%)	184 (30.2%)	286 (37.3%)	145 (28.4%)	
III	469 (24.9%)	163 (26.8%)	183 (23.9%)	123 (24.1%)	
IV	422 (22.4%)	136 (22.3%)	176 (23.0%)	110 (21.7%)	
					
GRADE OF DIFFERENTIATION					
Moderate	1696 (89.9%)	504 (82.7%)	717 (93.6%)	475 (93.1%)	<0.001
Poor	189 (10.1%)	105 (17.24%)	49 (6.39%)	35 (6.86%)	
HISTOLOGYC TYPE					
Adenocarcinoma	1687 (89.5%)	487 (80.0%)	712 (92.9%)	488 (95.7%)	<0.001
Mucinous	198 (10.5%)	122 (20.0%)	54 (7.1%)	22 (4.3%)	
EMERGENCY SURGERY	290 (15.4%)	101 (16.58%)	151 (19.71%)	38 (7.45%)	<0.001
PII	226 (11.9%)	69 (11.3%)	89 (11.6%)	68 (13.3%)	0.542

χ2 test was used to calculate the P‑values. PII, Postoperative Intraabdominal Infection

**Table 2:** Entire Cohort. Predictive factors of OS and CRS analyzed using Cox's proportional hazards model

	OS			CRS		
	HR	95% CI	P value	HR	95% CI	P value
TUMOR SITE						
RS (*)	1			1		
LS	0.697	0.511-0.902	0.042	1,092	0.864-1,303	0.574
Rectum	0.737	572-0.949	0.018	0.895	0.745-1.074	0.233
OBSTRUCTION	1.741	1.408-2.085	<0.001	1.731	1.404-2.135	<0.001
PERFORATION	2.197	1.711-2.821	<0.001	2,277	1,742-2,977	<0.001
T STAGE						
T1 (*)	1					
T2	5.552	3.377-9.128	<0.001	12,080	5.290-27.584	<0.001
T3	2.886	1.784-4.668	<0.001	5,650	2.500-12.771	<0.001
T4	2.160	1.275-3.658	0.004	3,377	1.416-8.055	0.006
N STAGE						
N0 (*)	1			1		
N1	3,921	3.248-4.733	<0.001	5.699	4.603-7.057	<0.001
N2	1,907	1.598-2.275	<0.001	2.580	2.102-3.167	<0.001
GRADE OF DIFFERENTIATION						
Well-Moderatelly Differentiated	0.800	0.651-0.981	0.032	0.791	0.637-0.984	0.035
AGE (years)						
≥ 70 (*)	1			1		
50-69	0.361	0.265-0.492	<0.001	0.425	0.308-0.586	<0.001
<50	0.508	0.437-0.591	<0.001	0.591	0.501-0.698	<0.001
PII	1,250	1.018-1.534	0.033	1.223	0.972-1.539	0.085

PII: Postoperative Intraabdominal Infection. HR: hazard ratio; 95% CI: 95% confidence interval; (*) Group of reference

**Table 3:** TNM Stage III and Stage IV. Predictive factors of OS and CRS analyzed using Cox's proportional hazards model

	OS			CRS		
STAGE III						
	HR	CI 95%	P value	HR	CI 95%	P value
TUMOR SITE						
RS (*)	1					
LS	0.991	0.686-1.433	0.963	0.932	0.630-1.380	0.725
Rectum	0.653	0.461-0.926	0.017	0.642	0.443-0.932	0.020
AGE (years)						
≥ 70 (*)	1			1		
50-69	0.428	0.245-0.745	0.003	0.542	0.305-0.961	0.036
<50	0.443	0.320-0.613	<0.001	0.534	0.379-0.752	<0.001
T STAGE						
T1 (*)	1			1		
T2	6.265	0.856-45,872	0.071	6.057	8.028-44.319	0.076
T3	2.664	0.386-19,263	0.332	2.319	0.321-16.757	0.404
T4	2.678	0.347-20,672	0.345	2.267	0.291-17.662	0.435
N STAGE						
N1 (*)	1			1		
N2	1.715	1.252-2.350	<0.001	1.803	1.288-2.525	<0.001
PERFORATION	0.650	0.364-1.160	0.145	0.548	0.307-0.980	0.042
						
STAGE IV (**)						
TUMOR SITE			0.012			0.020
RS (*)	1			1		
LS	0.679	0.511-0.902	0.007	0.691	0.520-0.918	0.011
Rectum	0.737	0.572-0.949	0.018	0.751	0.583-0.968	0.027
AGE (years)						
≥ 70 (*)	1			1		
50-69	0.364	0.230-0.576	<0.001	0.547	0.218-0.558	<0.001
<50	0.545	0.435-0.683	<0.001	0.349	0.436-0.687	<0.001
PERFORATION	1.586	1.088-2.313	0.016	1,606	1.101-2.343	0.014
T STAGE						
T2 (*)	1			1		
T3	1.541	0.711-3.340	0.273	1,522	0.702-3.299	0.287
T4	1.031	0.478-2.222	0.938	1,020	0.473-2.199	0.959
N STAGE						
N0 (*)	1			1		
N1	2.168	1.564-3.006	<0.001	2.215	1.593-3.079	<0.001
N2	1.312	0.936-1.841	0.115	1.327	0.943-1.866	0.105

HR: hazard ratio; 95% CI: 95% confidence Interval. (*) Group of reference. (**) In TNM Stage IV, the data reflect the risk of death 36 months after diagnosis.

**Table 4:** Entire cohort. Predictive factors of PRS analyzed using Cox's proportional hazards model

	HR	95% IC	P value
TUMOR SITE			
RS (*)	1		
LS	0.709	0.512-0.980	0.037
Rectum	0.644	0.475-0.875	0.005
T STAGE			
T1 (*)	1		
T2	9.039	2.693-30.331	<0.001
T3	4.364	1.298-14.670	0.017
T4	3.990	1.244-12.800	0.020
N STAGE			
N0 (*)	1		
N1	1.528	1.074-2.175	0.018
N2	1.332	0.997-1.778	0.052
PII			
AGE (years)			
≥ 70 (*)	1		
50-69	0.509	0.320-0.809	0.004
<50	0.509	0.387-0.670	<0.001

PII = Postoperative Intraabdominal Infection. HR: hazard ratio; 95% CI: 95% confidence interval. (*) Group of reference.

**Table 5:** Kaplan-Meier´s estimations of TTR, RFS and PRS according to the localization of primary tumor and Tumor Stage

	No. cases	Cases with Recurrence	TTR	median	P value	RFS	median	P value	PRS	median	P value
Entire Cohort											
RS	473	99	74	NR	0.188	63	NR	0.302	9	12	0.001
LS	589	122	76	NR		68	NR		13	25	
Rectum	399	105	70	NR		64	NR		22	26	
TNM I											
RS	126	3	96	NR	0.023	88	NR	0.351	25	11	0.401
LS	121	6	92	NR		84	NR		66	NR	
Rectum	132	14	86	NR		83	NR		37	36	
TNM II											
RS	184	26	79	NR	0.413	74	NR	0.227	14	16	0.227
LS	286	42	83	NR		72	NR		17	21	
Rectum	145	29	76	NR		66	NR		25	28	
TNM III											
RS	163	65	50	NR	0.205	43	44	0.159	6	10	0.002
LS	183	72	54	NR		50	NR		14	28	
Rectum	123	59	44	46		40	32		15	24	
