# Supplementary material for: Differences in Colorectal Cancer Survival Based on Primary Tumor Location: Retrospective Study from a Single Institution
Source: J Cancer. 2023 Aug 6;14(13):2444–54. doi: 10.7150/jca.85695 (PMC10475363; doi:10.7150/jca.85695)
Supplement: Supplementary file 1 — Supplementary tables. [file jcav14p2444s1.pdf]

SUPPLEMENTAL MATIRIAL

TABLE 1 S. Entire Cohort. Kaplan-Meier´s estimations of OS and CRS at 60 months after diagnosis categorized by patient´s and tumor characteristics

	No. cases	Cumulative OS at 60 months	median	P value	Cumulative CRS at 60 months	median	P value
**SEX**							
**Women**	731	56	NR	0.607	61	NR	0.575
**Men**	1154	53	NR		61	NR	
**PRIMARY TUMOR SITE**							
**RT**	609	49	NR	0.014	56	NR	0.020
**LT**	766	56	NR		62	NR	
**Rectum**	510	55	NR		62	NR	
**AGE (years)**							
**<50**	136	63	NR	0.001	64	NR	0.001
**50-69**	819	63	NR		66	NR	
**≥ 70**	930	45	51		55	NR	
**INTESTINAL OBSTRUCTION**							
**Si**	211	31	28	0.001	35	33	0.001
**No**	1674	57			64		
**PERFORATION**							
**Si**	107	24	22	0.001	35	25	0.001
**No**	1778	56			63		
**T STAGE**							
**T1**	180	88		0.001	95		0.001
**T2**	262	73			84		
**T3**	1087	54			61		
**T4**	356	24	21		26	23	
**N STAGE**							
**N0**	1068	70		0.001	81		0.001
**N1**	498	44	51		48	59	
**N2**	319	16	18		17	18	
**TNM STAGE**							
**I**	379	83	NR	0.001	93	NR	0.001
**II**	615	71	NR		82	NR	
**III**	469	52	NR		58	NR	
**IV**	422	8	18		9	18	
**GRADE OF DIFFERENTIATION**							
**Well-Moderate**	1696	57		0.001	63	NR	0.001
**Poor**	189	32	25		36	27	
**HISTOLOGYC TYPE**							
**Adenocarcinoma**	1687	55		0.001	62	NR	0.001
**Mucinous**	198	42	44		46	52	
**EMERGENCY SURGERY**	290	27	24	0.001	29	26	<0.001
**ELECTIVE SURGERY**	1595	61			62	NR	
**PII**							
**Yes**	226	49	NR	0.019	56	NR	0.062
**No**	1659	55	NR		61	NR	

The log-rank test was used to calculate P values. NR: not reached.

Table 2 S. TNM Stage I. Predictive factors of OS and CRS analyzed using Cox's proportional hazards model

	OS			CRS		
	**HR**	**95% IC**	**P value**	**HR**	**95% IC**	**P value**
**TUMOR SITE**						
**RS (*)**	1			1		
**LS**	1.299	0.681-2.476	0.427	2.984	0.800-11.127	0.104
**Rectum**	1.108	0.558-2.201	0.769	1.840	0.437-7.737	0.406
**AGE (years)**						
**≥ 70 (*)**				1		
**50-69**	<0.001	0.000	0.968	0.000	0.000	0.975
**<50**	0.395	0.216-0.720	0.002	0.501	0.184-1.366	0.177
**OBSTRUCTION**	41.555	4.605-374.973	0.001	73.238	6.500-825.159	0.001
**T STAGE**						
**T1 (*)**	1			1		
**T2**	1.846	1.039-3.280	0.037	2.045	0.720-5.811	0.179

HR: hazard ratio; 95% CI: 95% confidence interval. (*) Group of reference.

Table 3 S. TNM Stage II. Predictive factors of OS and CRS analyzed using Cox's proportional hazards model

	OS			CRS		
	**HR**	**95% IC**	**P value**	**HR**	**95% IC**	**P value**
**TUMOR SITE**			0.047			0.484
**RS vs LS**	1.338	0.866-0.068	0.190	1.004	0.538-1.873	0.990
**LS vs Rectum**	0.775	0.526-1.142	0.198	0.753	0.453-1.252	0.274
**AGE (years)**			<0.001			0.022
**≥ 70 vs 50-69**	0.195	0.071-0.533	0.001	0.270	0.083-0.874	0.029
**50-69 vs <50**	0.441	0.310-0.628	0.441	0.635	0.403-0.999	0.049
**OBSTRUCTION**	2.354	1.473-3.760	<0.001	2.901	1.601-5.256	<0.001
**PERFORATION**	3.924	2.417-6.373	<0.001	5.230	2.887-9.474	<0.001
**T STAGE**						
**T3 vs T4**	1.888	1.268-2.811	0.002	2.250	1.359-3.725	0.002
**GRADE OF DIFFERENTIATION**	0.628	0.384-1.211	0.192	0.592	0.285-1.232	0.161
**PII**	2.079	1.425-3.034	<0.001	2.811	1.740-4.541	<0.001

PII: Postoperative Intraabdominal Infection

TABLE 4 S. TNM Stage I,II, and III. Kaplan-Meier´s estimations of RFS and PRS at 60 months after diagnosis categorized by patient´s and tumor characteristics

	No. Patients	Patients with recurrence	Cumulative RFS at 60 months (%)	median	P value	Cumulative PRS at 60 months (%)	median	P value
**SEX**								
**Women**	554	109	77	NR	0.072	15%	24	0.952
**Men**	907	217	72	NR		16%	23	
**PRIMARY TUMOR**								
**SITE**	473	99	76	NR	0.159	10	15	0.006
**RT**	589	122	75	NR		14	27	
**LT**	399	105	70	NR		22	26	
**Rectum**								
**AGE (years)**					0.374			<0.001
**<50**	102	27	74	NR		16	25	
**50-69**	631	137	75	NR		22	33	
**≥ 70**	728	162	72	NR		11	15	
**INTESTINAL**								
**OBSTRUCTION**					0.020			0.346
**No**	1339	293	75	NR		16	23	
**Yes**	122	33	63	NR		12	22	
**PERFORATION**					<0.001			0.009
**No**	1391	301	75	NR		17	24	
**Yes**	70	25	50	NR		6	11	
**T STAGE**					<0.001			0.002
**T1**	180	9	92	NR		66	-	
**T2**	251	27	87	NR		25	24	
**T3**	857	210	71	NR		15	24	
**T4**	173	80	45			9	14	
**N STAGE**					<0.001			0.007
**N0**	993	130	84	NR		24	26	
**N1**	357	132	56	NR		12	23	
**N2**	111	64	33	23		7	15	
**GRADE OF**								
**DIFFERENTIATION**					<0.001			0.006
**Well-Moderate**	1356	288	75	NR		16	24	
**Poor**	105	38	59	NR		13	8	
**HISTOLOGYC TYPE**					0.770			0.004
**Adenocarcinoma**	1329	286	74	NR		16	24	
**Mucinous**	132	40	69	NR		14	8	
**Cirugia Urgente**	158	44	61	NR	0.001	8	7	0.009
**Cirugía Programada**	1303	282	75	NR		17	24	
**PII**					0.001			0.624
**No**	1271	273	75	NR		15	23	
**Yes**	190	53	64	NR		18	24	

The log-rank test was used to calculate P values. NR: not reached. PII: Postoperative Intraabdominal Infection
